# Cardioprotective Properties of Humoral Factors Released after Remote Ischemic Preconditioning in CABG Patients with Propofol-Free Anesthesia—A Translational Approach from Bedside to Bench

**DOI:** 10.3390/jcm11051450

**Published:** 2022-03-07

**Authors:** Katharina Feige, Carolin Torregroza, Milena Gude, Patrick Maddison, Martin Stroethoff, Sebastian Roth, Giovanna Lurati Buse, Markus W. Hollmann, Ragnar Huhn

**Affiliations:** 1 Department of Anesthesiology, University Hospital Duesseldorf, Medical Faculty, Heinrich-Heine-University Duesseldorf, Moorenstr. 5, 40225 Duesseldorf, Germany; katharinakristina.feige@med.uni-duesseldorf.de (K.F.); milena.gude@hhu.de (M.G.); patrick.maddison@hhu.de (P.M.); martin.stroethoff@med.uni-duesseldorf.de (M.S.); sebastian.roth@med.uni-duesseldorf.de (S.R.); giovanna.luratibuse@med.uni-duesseldorf.de (G.L.B.); ragnar.huhn@med.uni-duesseldorf.de (R.H.); 2 Department of Anesthesiology, Amsterdam University Medical Center (AUMC), Meiberdreef 9, 1105 AZ Amsterdam, The Netherlands; m.w.hollmann@amsterdamumc.nl; 3 Department of Anesthesiology, Kerckhoff-Clinic GmbH, Benekestr. 2-8, 61231 Bad Nauheim, Germany

**Keywords:** remote ischemic preconditioning, cardioprotection, reperfusion injury, diabetes mellitus

## Abstract

The cardioprotective effect of remote ischemic preconditioning (RIPC) is well detectable in experimental studies but not in clinical trials. Propofol, a commonly used sedative, is discussed to negatively influence the release of humoral factors after RIPC. Further, results from experimental and clinical trials suggest various comorbidities interact with inducible cardioprotective properties of RIPC. In the present study, we went back from bedside to bench to investigate, in male patients undergoing CABG surgery, whether (1) humoral factors are released after RIPC during propofol-free anesthesia and/or (2) DM interacts with plasma factor release. Blood samples were taken from male patients with and without DM undergoing CABG surgery before (control) and after RIPC (RIPC). To investigate the release of cardioprotective humoral factors into the plasma, isolated perfused hearts of young rats (*n* = 5 per group) were used as a bioassay. The hearts were perfused with patients’ plasma without (Con) and with RIPC (RIPC) for 10 min (1% of coronary flow) before global ischemia and reperfusion. In additional groups, the plasma of patients with DM was administered (Con DM, RIPC DM). Infarct size was determined by TTC staining. Propofol-free RIPC plasma of male patients without DM showed an infarct size of 59 ± 5% compared to 61 ± 13% with Con plasma (*p* = 0.973). Infarct sizes from patients with DM showed similar results (RIPC DM: 55 ± 3% vs. Con DM: 56 ± 4%; *p* = 0.995). The release of humoral factors into the blood after RIPC in patients receiving propofol-free anesthesia undergoing CABG surgery did not show any cardioprotective properties independent of a pre-existing diabetes mellitus.

## 1. Introduction

Causal therapy of myocardial injury (MI) in the form of immediate restoration of coronary blood circulation remains the gold standard for patients suffering from MI [1]. Paradoxically, the resupply of coronary blood flow itself provokes ischemia-reperfusion injury (I/R), restraining the outcome of the reperfusion-intervention [2].

Establishing feasible and ideally minimally invasive approaches for perioperative cardioprotection against I/R injury has been the subject of research for some time. In the experimental setting, conditioning strategies induced ischemically (e.g., remote ischemic preconditioning (RIPC)) or pharmacologically have been shown to cause strong cardioprotection [3]. Unfortunately, translation of these experimental strategies into the clinical routine is still challenging and has yet failed to be successful [4]; considering the majority of large clinical trials demonstrate no detectable benefit for patient’s outcome [5,6]. The lack of transferability is partly caused by the invasiveness of ischemic-induced stimuli, which is only attainable in cardiac surgery or during interventional therapy. However, the divergence between experimental and clinical results cannot solely be explained by the applied conditioning approach. More confounding factors need to be considered—specifically patients’ comorbidities, e.g., diabetes mellitus (DM), comedication, age or anesthetic regime [7,8].

A promising non-invasive and well-investigated approach is RIPC—described as short cycles of transient ischemia and reperfusion of peripheral tissue [9]. The remote ischemic stimulus provokes the release of endogenous cardioprotective factors, increasing the tolerance of the myocardial tissue against a subsequent I/R injury [10,11]. In contrast to direct ischemic conditioning interventions, RIPC is less invasive and, therefore, easy, feasible and more practicable in patients. While results from animal studies on RIPC were convincing, findings from clinical trials were inconsistent. ERICCA [6] and RIPHeart [5], two large randomized multi-center trials including over 1000 patients each, did not show any effect of RIPC on patient’s outcome undergoing heart surgery. As in both studies, nearly all of the patients were anesthetized, applying propofol; it was assumed that propofol might abrogate the release of humoral factors after RIPC. This hypothesis was further investigated and confirmed in experimental and clinical studies [12,13].

However, while propofol has been clearly demonstrated to hamper conditioning strategies in the clinical setting, more of the above-mentioned influencing factors need to be regarded in the context of successful translation of cardioprotection. One of the most common comorbidities associated with coronary heart disease—and thus frequently seen in patients undergoing cardiac surgery—is diabetes mellitus [14]. Experimental studies have already demonstrated hyperglycemia and diabetes mellitus to fully block ischemic and pharmacological conditioning strategies in vivo and in vitro [15,16,17,18]. Hence, diabetes appears to be a relevant confounding factor to be regarded in the challenge of clinical cardioprotection.

Therefore, with this translational approach back from bedside to bench, we aim to answer the question of whether RIPC under propofol-free anesthesia in patients undergoing CABG surgery leads to the release of cardioprotective humoral factors to the plasma and further if humoral factors are released in patients suffering from DM. In order to discriminate between the release of humoral factors and possible other confounders, we employed a translational approach transferring plasma sampled in vivo onto isolated, young, healthy hearts in vitro.

## 2. Materials and Methods

All experiments were performed in accordance with the Guide for the Care and Use of Laboratory Animals published by the U.S. National Institute of Health (NIH publication No. 85-23, revised 1996) after approval by the local Animal Care and Use Committee of the University of Duesseldorf (project number O27/12).

### 2.1. RIPC in Patients

After written informed consent, blood samples were taken from male patients undergoing CABG surgery in vivo with and without DM. Criteria for inclusion and exclusion are listed in Table 1.

**Figure 1:**
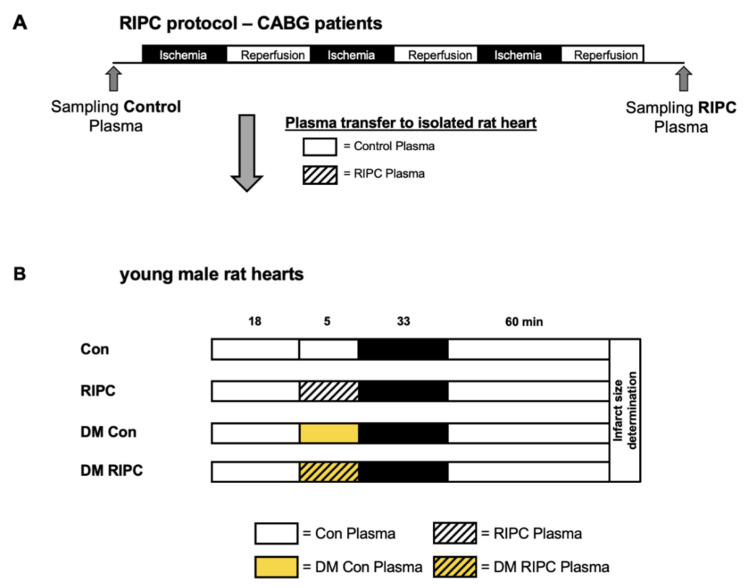
Experimental protocol and plasma transfer. (**A**): RIPC protocol for plasma sampling in CABG patients. (**B**): Transfer of plasma to young male rat hearts. CABG = Coronary Artery Bypass Graft; Con = Control plasma, RIPC = Remote Ischemic Preconditioning plasma; DM Con = Diabetes mellitus Control plasma; DM RIPC = Diabetes mellitus Remote Ischemic Preconditioning plasma.

Anesthesia was induced with etomidate, sufentanil and rocuronium and maintained with the combination of a volatile anesthetic (sevoflurane or desflurane) and continuous administration of sufentanil or remifentanil. Etomidate was used to guarantee propofol-free anesthesia in order to exclude a single induction dose of propofol that might influence the effect of RIPC. As shown in Figure 1A, after the induction of anesthesia, RIPC was induced by three cycles of 5 min of ischemia of one upper arm, each followed by 5 min of reperfusion. Ischemia was induced by inflating a blood pressure cuff to 200 mmHg and reperfusion started by deflating the cuff. A total of 50 mL whole blood was taken (lithium heparin for anticoagulation) from the cubital vein of the opposite arm 5 min before and after RIPC (control and RIPC, respectively). Plasma was separated by centrifugation and was stored at −80 °C until further use.

### 2.2. Surgical Preparation

All in vitro experiments were performed on isolated hearts of young (aged 2–3 months) male Wistar rats. The animals were housed on a 12:12 h light/dark schedule with free, unlimited access to standard chow and water. As we have described previously [19], rats were anesthetized by intraperitoneal injection of pentobarbital (80 mg/kg body weight) and thoracotomized for resection of the heart. As excised, the hearts were mounted immediately on a Langendorff system and were perfused with Krebs–Henseleit solution containing (in mM) 118 NaCl, 4.7 KCl, 1.2 MgSO_4_, 1.2 KH_2_PO_4_, 25 NaHCO_3_, 0.5 EDTA, 2.25 CaCl_2_, 11 glucose and 1 lactate, at 37 °C and constant pressure of 80 mmHg. For recording hemodynamic parameters, a small fluid-filled balloon was placed into the left ventricle, and the left ventricular end-diastolic pressure (LVEDP) was set at 2–8 mmHg. Throughout experiments, heart rate, left ventricular pressure (LVP) and coronary flow were measured continuously and digitized using an analog to digital converter (PowerLab/8SP, ADInstruments Pty Ltd., Castle Hill, Australia) at a sampling rate of 500 Hz. The left ventricular developed pressure (LVDP) was calculated as a maximal left ventricular systolic pressure (LVSP)–left ventricular end-diastolic pressure (LVEDP). As a further indicator for myocardial damage, maximal contracture during ischemia, as well as the respective time-point, was analyzed for each experiment. The data were continuously recorded on a personal computer using Chart for Windows v5.0 (ADInstruments Pty Ltd., Castle Hill, Australia).

### 2.3. Experimental Protocol

The experimental protocol is shown in Figure 1. All hearts underwent an equilibration period of 20 min. The hearts were randomly assigned to an experimental group (*n* = 5 per group). Prior to ischemia, the hearts were perfused with undiluted patient plasma for 10 min at a perfusion rate of 1% of the coronary flow. Subsequently, the hearts were subjected to global ischemia of 33 min, followed by 60 min of reperfusion.

Control group (Con): Hearts were perfused with plasma taken from CABG patients without RIPC treatment.
Remote Ischemic Preconditioning group (RIPC): Hearts were perfused with plasma taken from CABG patients with RIPC treatment.
Diabetes mellitus + Control group (DM Con): Hearts were perfused with plasma taken from CABG patients with DM but without RIPC treatment.
Diabetes mellitus + RIPC group (DM RIPC): Hearts were perfused with plasma taken from CABG patients with DM and with RIPC treatment.

At the end of the reperfusion, the hearts were frozen at −20 °C for later infarct size staining. The frozen hearts were cut into 8 transverse 1 mm thick slices and stained in 0.75% 2,3,5-triphenyltetrazolium chloride (TTC) for 15 min at 37 °C. Subsequently, slices were fixed in 3.75% paraformaldehyde for 2 h. The fixated slices were scanned, and infarct size was analyzed by computerized planimetry (Sigma Scan Pro, Version 5, SYSTAT Software, San Jose, CA, USA). The infarct size was calculated as a percentage of the left ventricle.

### 2.4. Statistical Analysis

#### 2.4.1. Sample Size Analysis

Calculation of sample size was performed by using GraphPad StatMate™ (GraphPad Software, San Diego, CA, USA) and resulted in a group size of n = 5 for detecting a 25% mean difference in infarct size with a power of 80% (α < 0.05 (two-tailed)).

#### 2.4.2. Statistical Approach

To compare hemodynamic variables between groups or between different time points within groups, we used a two-way analysis of variance (ANOVA) and a Tukey post hoc test (GraphPad Software, San Diego, CA, USA). An investigator blinded to the experimental groups determined the infarct sizes. A one-way ANOVA was chosen, followed by a Tukey post hoc test to analyze infarct size. Data are presented as mean ± SD. Differences were regarded statistically significant when *p* < 0.05.

## 3. Results

### 3.1. Patient and Animal Characteristics

Patient characteristics are shown in Table 2. The study showed no differences in animal characteristics (Table 3).

### 3.2. Infarct Size Analysis

**Figure 2:**
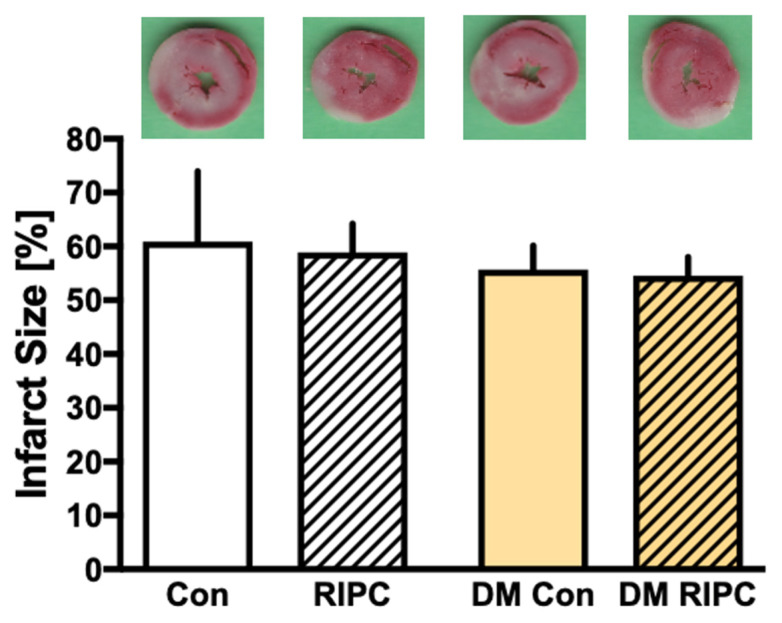
Infarct size measurement. Histogram showing all infarct sizes of the study. Con = Control plasma; RIPC = Remote Ischemic Preconditioning plasma; DM Con = Diabetes mellitus Control plasma; DM RIPC = Diabetes mellitus Remote Ischemic Preconditioning plasma. Data are presented as means ± SD.

The infarct sizes and representative heart slices of the respective group are shown in Figure 2. Preconditioning with RIPC plasma from male CABG patients without DM (RIPC) did not reduce infarct size significantly compared to hearts treated with Con plasma of CABG patients without DM (RIPC: 59 ± 5% vs. Con: 61 ± 13%; *p* = 0.973). The transferred plasma of CABG patients with DM (DM Con) showed similar results. The infarct size of naive hearts treated with RIPC DM plasma was 55 ± 3%. The administration of DM Con plasma showed an infarct size of 56 ± 4% (*p* = 0.995 vs. DM RIPC).

### 3.3. Cardiac Function

The hemodynamic data are demonstrated in Table 4. There were no statistical differences measured in heart rate, LVDP and coronary flow between the groups at baseline and during ischemia and reperfusion. After ischemia and during reperfusion, LVDP and coronary flow were statistically decreased compared to baseline within each group.

## 4. Discussion

The main findings of our current study demonstrate that RIPC in patients undergoing CABG surgery with propofol-free anesthesia does not confer cardioprotection and further that the absence of infarct size reduction was independent of an existing DM in these patients.

RIPC is considered a promising non-invasive method to protect the heart against I/R injury and, therefore, is seen to be an ideal protective measure, especially for clinical use [20]. However, its clinical implementation remains difficult as data from clinical trials are contradictory. Thielmann et al. [21] demonstrated significantly lower troponin levels, a lower rate of major adverse cardiac and cerebrovascular events in patients undergoing RIPC treatment 72 h after CABG surgery. Hoole and colleagues showed similar effects of RIPC in patients undergoing percutaneous coronary interventions (PCI) [22]. However, the two large, randomized multi-center trials, ERICCA and RIPHeart, failed to show any effect of RIPC on the clinical outcome of cardiac surgery patients [23]. 

Anesthetic regimen, especially the application of propofol, has been discussed in detail as a limitation for obstructing the cardioprotective effects of RIPC in experimental and clinical trials. Our own experimental study showed—in vivo and in vitro—that propofol blocked the release of humoral factors during RIPC but had no effect on RIPC-induced cardioprotection once protective humoral factors were released to the plasma [12]. Kottenberg et al. [13] demonstrated that an anesthetic regime with propofol abolished cardioprotection in CABG patients, whereas the effect was maintained with the volatile anesthetic isoflurane. The results showed a significant reduction in troponin values at 72 h postoperative by RIPC during isoflurane compared to propofol anesthesia. Interestingly, our study demonstrates that RIPC plasma taken after propofol-free anesthesia induction from aged non-diabetic patients undergoing CABG surgery does not induce cardioprotective properties. These contrary findings might be caused by a different study setup (clinical in vivo vs. experimental in vitro), primary endpoints (troponin values vs. infarct size) or other influencing factors, such as different comorbidities in the respective patients.

It remains undetermined whether a possible loss of protection by RIPC under confounding factors, such as diabetes mellitus, could be caused by obstruction—either the release or the transfer—of humoral factors into the bloodstream, subsequently activating known myocardial signaling cascades [10]. The results from our current study show that RIPC plasma from patients undergoing CABG surgery, even with propofol-free anesthesia, does not induce cardioprotection, and these findings were independent of pre-existing diabetes mellitus. However, the underlying cause of blocked infarct size reduction cannot be determined definitely, and further investigations are needed. It is conceivable that a variety of confounding patient factors might be responsible for the lack of cardioprotection in our experimental setup. Next to the anesthetic regimen and diabetes mellitus, other comorbidities, e.g., hypertension, medications, age or sex, are discussed [7,24]. Patient characteristics, such as age, sex and medication, have been demonstrated to influence humoral factor release after RIPC [11]. In our current study, we only included plasma from male patients undergoing CABG surgery. This approach eliminates the potential influence of sex on our results in the context of cardioprotection by RIPC. Further, all patients being treated with sulfonylureas were excluded from the study. The modification of the ATP-sensitive potassium channel (mK_ATP_) by sulfonylureas seems to be an integral player of the RIPC stimulus releasing a circulating effector via local neurogenic mechanisms [25,26].

Aging was shown to block cardioprotective conditioning strategies in experimental studies. In an experimental in vivo rat study, we demonstrated that infarct size reduction of RIPC was abolished in the aged rat heart, whereas cardioprotection was fully present in the young rat [27]. Further, in human volunteers, we showed an age-dependent and sex-specific effect of RIPC on humoral factor release [28]. RIPC plasma from young male volunteers induced a strong infarct size reduction, whereas plasma from aged male volunteers was not protective. The set up for plasma extraction in volunteers and the transfer to the in vitro rat heart was the same as in the present study. Interestingly, transferring RIPC plasma from young male volunteers to an aged rat heart still led to a significant infarct size reduction. Our findings indicate a possible lack of protective humoral factor release to the aged subject after RIPC. These results are comparable to the above-mentioned blocking effects of propofol. Both advanced age and the anesthetic regimen seem to inhibit factor release after RIPC. Considering this knowledge, one conceivable explanation for the lack of infarct size reduction by RIPC plasma in our current study might be the advanced age (shown in Table 1) of the respective patients.

The results of the present study must be interpreted in light of several study limitations. First, a possible negative effect of etomidate on factor release after RIPC cannot be ruled out by our experimental protocol. To this point, little research has been done on the influence of etomidate on cardioprotection; however, so far, no inhibiting effects have been described for induction with etomidate on conditioning strategies. Additionally, we did not unravel the underlying and potentially blocked intracardiac signaling mechanisms of RIPC under propofol-free anesthesia with or without diabetes mellitus. The aim of the study was to focus on the presumed confounding factors, propofol and diabetes mellitus, discussed in clinical trials to abolish the protective effects of RIPC. To our knowledge, this is the first study addressing the question of RIPC-mediated cardioprotection employing a translational approach with plasma from human volunteers undergoing propofol-free anesthesia.

## 5. Conclusions

The results of our study demonstrate that RIPC in patients undergoing CABG surgery with propofol-free anesthesia does not confer cardioprotection. Furthermore, the absence of infarct size reduction was independent of a pre-existing DM in these patients. Our results indicate that propofol might not be the only influencing factor on RIPC-induced cardioprotection. More research is needed to evaluate the remaining challenge of translating this intervention into clinical trials.

## Figures and Tables

**Table 1:** Criteria for inclusion and exclusion.

Inclusion	- written informed consent - indication for CABG surgery with or without HLM - Diabetes mellitus II - normal performance of upper limbs
Exclusion	- missing consent - age < 18 years - medication with sulfonylureas - emergency surgery - repeated interventions - pre-existing assist devices (Impella^®^, LVAD, ECLS, IABP) - pre-existing nerve damage of the upper limb - status after thrombo-embolic events - chronic pain disorders and psychiatric or neurologic - disorders leading to missing legal competence

HLM = Heart Lung Machine; LVAD = Left Ventricular Assist Device, ECLS = Extra Corporal Life Support system; IABP = Intraaortal Balloon Pump.

**Table 2:** Patient characteristics.

Demographics	Non-Diabetic	Diabetic
Male (*n*)	10	10
Age (years)	72 ± 13	65 ± 8
Height (cm)	175 ± 6	180 ± 5
Weight (kg)	84 ± 15	93 ± 18
BMI	27 ± 4	29 ± 5
**Surgical procedure**	***n***	
OPCAB	2	6
MIDCAB	2	0
ACB on-pump	6	4
**Risk factors/comorbidities**	***n***	
Diabetes mellitus	0	10
Hypertension	6	8
Hyperlipidemia	7	7
Chronic obstructive pulmonary disease	4	2
Peripheral vascular disease	3	0
Chronic kidney disease	3	0
**Medication**	***n***	
Aspirin	6	8
ADP-Receptor Antagonists	4	2
Statins	6	8
Beta-blockers	7	7
ACE inhibitors	4	4
Anti-diabetics (except sulfonylureas)	0	10

Patient characteristics are mean ± standard deviation; surgical procedures, risk factors and medication are absolute numbers. BMI = Body Mass Index; OPCAB = Off-Pump Coronary Artery Bypass; MIDCAB = Minimal Invasive Direct Coronary Artery Bypass; ACB = Aorto-Coronary Bypass.

**Table 3:** Animal weights and ischemic contracture.

	*n*	Body Weight (g)	Heart Weight Wet (g)	Time of Max. Ischemic Contracture (min)	Level of Max. Ischemic Contracture (mmHg)
Con	5	299 ± 26	1.18 ± 0.05	17 ± 1	62 ± 5
RIPC	5	292 ± 9	1.15 ± 0.09	17 ± 2	58 ± 1
Con DM	5	301 ± 13	1.24 ± 0.03	16 ± 2	66 ± 8
RIPC DM	5	299 ± 19	1.21 ± 0.06	15 ± 1	65 ± 5

Data are mean ± SD. Con = Control; RIPC = Remote Ischemic Preconditioning; DM = Diabetes mellitus.

**Table 4:** Hemodynamic variables.

	Baseline	PC	Reperfusion	
			**30**	**60**
Heart Rate (bpm)				
Con	271 ± 54	252 ± 65	197 ± 81	252 ± 49
RIPC	295 ± 15	285 ± 20	279 ± 43	219 ± 71
Con DM	292 ± 10	278 ± 27	254 ± 27	237 ± 9
RIPC DM	308 ± 24	288 ± 28	295 ± 42	282 ± 36
Left Ventricular Developed Pressure (mmHg)				
Con	131 ± 32	114 ± 33	23 ± 12 *	21 ± 13 *
RIPC	142 ± 14	123 ± 40	30 ± 13 *	33 ± 9 *
Con DM	136 ± 18	116 ± 18	17 ± 13 *	22 ± 10 *
RIPC DM	111 ± 22	104 ± 39	29 ± 14 *	34 ± 10 *
Coronary flow (mL/min)				
Con	16 ± 2	12 ± 4	10 ± 2 *	10 ± 3 *
RIPC	16 ± 2	15 ± 4	9 ± 3 *	11 ± 5 *
Con DM	16 ± 3	12 ± 4	8 ± 1 *	9 ± 2 *
RIPC DM	13 ± 4	13 ± 3	9 ± 1 *	8 ± 1 *

Data are mean ± SD. Con = Control; RIPC = Remote Ischemic Preconditioning; DM = Diabetes mellitus. * *p* < 0.05 versus baseline.

## Data Availability

Not applicable.
